# Atypical dengue fever in a partially vaccinated patient: a case report

**DOI:** 10.1590/S1678-9946202567076

**Published:** 2025-11-03

**Authors:** Vasco João Mendes, Ezequias Batista Martins, Otilia Lupi, Anielle de Pina-Costa, Guilherme Amaral Calvet, Clarisse da Silveira Bressan, Ana Beatriz T. B. C. Ferreira, Fernanda de Bruycker-Nogueira, Ana Maria Bispo Filippis, Patrícia Brasil

**Affiliations:** 1 Fundação Oswaldo Cruz, Instituto Nacional de Infectologia Evandro Chagas, Rio de Janeiro, Rio de Janeiro, Brazil; 2 Fundação Oswaldo Cruz, Instituto Nacional de Infectologia Evandro Chagas, Laboratório de Doenças Febris Agudas, Rio de Janeiro, Rio de Janeiro, Brazil; 3 Fundação Oswaldo Cruz, Instituto Oswaldo Cruz, Laboratório de Flavivírus, Rio de Janeiro, Rio de Janeiro, Brazil

**Keywords:** Dengue, Vaccine, Immunologic factors, Protection

## Abstract

Dengue fever is an acute, systemic, and debilitating febrile illness that poses a significant global public health threat. Vaccination is important in combating the virus in highly prevalent countries, as it reduces the risk of symptomatic infection, hospitalizations, morbidity, and mortality. We report a unique case of atypical dengue fever in a previously healthy 42-year-old Brazilian woman. She developed dengue without the characteristic fever or elevated inflammatory markers 15 days after her initial TAK-003 (Q-denga) vaccine dose, setting her case apart from typical manifestations. ’Whether the mildness of the case was due to the vaccine’s protective effect or if it was caused by the vaccine virus itself, as genetic sequencing of DENV-2 was not possible, is unclear. In regions where the vaccine is being introduced, atypical cases, particularly those without fever, require thorough investigation, so dengue can be excluded.

## INTRODUCTION

Dengue fever is an acute febrile illness with clinical manifestations ranging from asymptomatic to severe clinical types that can lead to multiple organ dysfunction. This is a serious public health problem worldwide, and has lethal potential^1^. Globally, dengue is regarded as the most relevant mosquito-borne viral disease^2^.

The disease is hyperendemic in tropical and subtropical countries, primarily in urban and semi-urban areas. The global incidence of dengue has grown exponentially recently, with almost half of the world’s population now at risk of infection^1^. Dengue virus (DENV) is a flavivirus belonging to the *Flaviviridae* family, it has a single-stranded RNA genome and comprises three structural proteins (C - capsid, E - envelope, and M - membrane)^3^. There are four serotypes of dengue virus (DENV-1, 2, 3, and 4). DENV-1 isolates belong to genotype V, DENV-2 to the Asian-American genotype, DENV-3 to genotypes I and III, and DENV-4 to genotypes I and II^2^.

After infection with a specific DENV serotype, permanent specific immunity is acquired against that serotype, but not against others. Reinfection with a different serotype may result in a more severe disease presentation^1^. The first epidemic in Brazil occurred in 1981, followed by a rapid expansion of dengue fever over the years, with all four serotypes of the virus now circulating simultaneously. There are transmitting mosquitoes throughout all regions of the country^2^. In 2024, 10 million probable cases of dengue fever and approximately 5,000 deaths were reported in the Americas, and Brazil had the highest number of cases—with more than 400 cases per 100,000 inhabitants in 2024^4^. As no antiviral medication is currently approved for dengue fever, treatment remains symptomatic and relies on clinical support. Laboratory tests are necessary for monitoring the clinical course of the disease from the third day of symptoms until recovery^5^.

The development of a safe tetravalent vaccine that produces a balanced immune response to all four serotypes has been a longstanding goal, and its most promising approach involves using a live attenuated virus. Despite numerous challenges, the development of dengue vaccine candidates has continued steadily over the past decade. However, effective dengue control requires a comprehensive approach that integrates vaccination with strategic interventions including vector control, educational programs, treatment of severe cases, and rigorous epidemiological surveillance^5^.

CYD-TDV (Dengvaxia) is a recombinant, live-attenuated tetravalent vaccine licensed in more than 20 countries. In a randomized study of this vaccine, involving participants aged two to 16 years, the incidence of symptomatic dengue during the acute phase was lower in the vaccinated group compared to the placebo group. The efficacy estimates ranged from 77.4% to 89.2%. For participants aged nine to 16 years, it ranged from 78.7% to 92.1%. Overall protective efficacy against all four serotypes was confirmed in Asia; however, this vaccine caused many deaths in children who had never had dengue. Therefore, it has the limitation of only being given to those who are DENV-IgG positive^6^.

The Butantan-DV vaccine is a Brazilian tetravalent live-attenuated virus vaccine. In a phase 3 clinical trial, healthy participants (aged two to 59) who had not previously received dengue vaccine were included’, and its efficacy against any dengue serotype was 67.3%. However, this vaccine is not yet available^7^.

TAK-003 (Qdenga^®^) is a tetravalent vaccine (attenuated virus) produced with four DENV strains: one attenuated DENV-2 strain and three recombinant DENV-2 strains. It also contains the membrane and envelope genes of DENV-1, DENV-3, and DENV-4 serotypes^8^. Studies have demonstrated that TAK-003 is immunogenetically well tolerated and effective in adults, from non-endemic regions, with no prior exposure to DENV. It has also shown efficacy in both adults and children in endemic areas across Asia and Latin America^8^. After four years, the study confirmed that the cumulative efficacy of the vaccine against symptomatic dengue was 61.2%, and the efficacy against dengue leading to hospitalization was 84.1%^9^.

Here, we show a case of an adult woman who had atypical manifestations 15 days after receiving the TAK-003 vaccine, raising doubts about reactions produced by a vaccine strain or whether a single dose of vaccine is capable of producing a good immune response.

### Ethics

The study was approved by the research ethics committee of the Instituto Nacional de Infectologia Evandro Chagas, Fiocruz, under the process Nº CAAE 88551218.6.0000.5262.

## CASE REPORT

A previously healthy 42-year-old Brazilian woman, born and resident in Rio de Janeiro, with no history of prior DENV infection, received the first dose of the TAK-003 vaccine on May 26, 2024.

**Figure 1:**
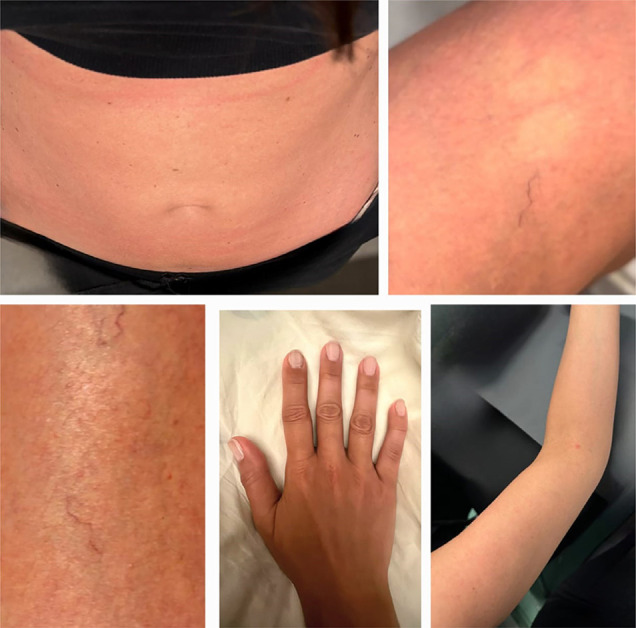
Extensive maculopapular rash and moderate arthritis on the patient’s right hand.

On June 9, 2024 (15 days after vaccine administration), the patient experienced severe headache and moderate prostration. On the second day (June 10, 2024), she reported dysesthesia in the upper limbs, maculopapular rash (upper limbs, chest, and abdomen), and moderate pruritus. On the third day (June 11, 2024), the rash spread throughout her entire body (Figure 1), accompanied by intense pruritus and myalgia in the abdominal muscles. On the fourth day (June 12, 2024), she developed moderate arthralgia in both hands, and moderate arthritis appeared in the second right finger. Notably, the patient did not experience fever at any point during the illness.

A physical examination and medical evaluation were performed on the second day of symptoms, revealing two discrete, non-tender lymph nodes (retro auricular and cervical). Vital signs and the remainder of the physical examination were unremarkable. She had no warning signs.

A follow-up medical evaluation was conducted eight days later (June 17, 2024). Physical examination revealed a maculopapular rash distributed throughout the body, two palpable lymph nodes in the left occipital region (non-painful), moderate right ankle edema, and slight walking difficulty. The condition persisted for ten days, with a complete resolution of symptoms occurring on June 18, 2024.

Hematology, blood biochemistry, and serological tests were performed on the second and ninth days after symptoms onset. Hematology and biochemistry results remained within normal limits. Serum samples tested positive for anti-DENV-IgM, and real-time reverse transcriptase polymerase chain reaction (rRT-PCR) detected DENV serotype 2 on the second day of symptoms. The test revealed a high cycle threshold (Ct) value (very close to control values), indicating a low viral load, making viral amplification impossible to confirm if the strain was wild or vaccine. rRT-PCR tests did not detect Zika or chikungunya in serum, urine, and saliva samples. Serology for chikungunya was also negative. Seroconversion of anti-DENV-IgG was observed one month after symptoms onset, along with disappearance of anti–DENV-IgM. Table 1 summarizes the results of all tests performed.

**Table 1:** Laboratory tests performed

	2024 June 10	2024 June 17	2024 August 12
Haemoglobin (g/dL)	13.3	13.7	---
Haematocrit (%)	38.8	41.1	---
White blood cells (x10^3^/µL)	7,360	10,480	---
Platelets (x10^3^/µL)	236,000	338,000	---
Alanine aminotransferase (IU/L)	51	51	---
Aspartame aminotransferase (UI/L)	29	33	---
Alkaline phosphatase (UI/L)	113	107	---
Gamma-glutamyl transferase (U/L)	248	246	---
Total bilirrubina (mg/dL)	0.37	0.28	---
Direct bilirrubun (mg/dL)	0.08	0.08	---
Indirect billirrubin (mg/dL)	0.29	0.19	---
Albumin (g/dL)	3.7	4.08	---
Blood urea nitrogen (mg/dL)	24	28	---
Creatinine (mg/dL)	0.73	0.76	---
Erythrocyte sedimentation rate (ESR) (mm/hr)	5	15	---
C-reactive protein (CRP) (mg/dL)	0.16	0.15	---
DENV IgM - ELISA	Positive	Positive	Negative
DENV IgG - ELISA	Negative	Negative	Positive
NS1	Negative	Negative	---
RT-PCR (Serum) - Dengue	Detected (DENV 2)	Not detected	---

## DISCUSSION

In this case report, we show an atypical clinical presentation of dengue fever, with no warning signs, developed 15 days after administration of the first dose of the TAK-003 vaccine. The patient did not experience fever, and the clinical presentation would not be included among the differential diagnosis for acute febrile illness.

After receiving the first vaccine dose, the patient may have been infected by a wild DENV-2, leading to an atypical and mild clinical presentation, characterized by the absence of fever and normal inflammatory markers (ESR and CRP). This mild response is plausible, as a single dose of the vaccine can elicit a high to very high immunogenic response to all DENV serotypes among adults (≥70%) and children/adolescents (≥90%)^10^. Our patient also demonstrated positive anti–DENV–IgM on the second day after symptom onset, indicating an excellent early immunological response. This occurred regardless of whether the stimulation was from a wild or vaccine strain. Conversely, the clinical manifestations could also be attributed solely to the vaccine virus—either an infection or an allergic reaction.

However, the presence of vaccine viremia starts in the second week after vaccination and persists for up to 30 days, without symptoms, as already demonstrated in a study on safety and immunogenicity of the TAK-003 vaccine^11^. The patient experienced constitutional symptoms and early rash, differing from typical vaccine viremia.

For the reported case, regardless of the origin of the strain (wild or viral), we have an atypical presentation of the disease, resulting from low viremia. Here, RT-PCR for DENV performed on the second day of symptoms revealed a high Ct value, demonstrating low viremia, which limited RNA amplification. Bhatt *et al*.^12^ describe that the cycle threshold (Ct) of 40 copies/ml for RT-PCR would be the maximum threshold for generating reliable detection results. Numerous “in vitro” and “in vivo” studies have been conducted worldwide to investigate the role of dengue viremia in disease pathogenesis and severity, but the results have been inconsistent. Additionally, there is limited literature on the kinetics of dengue viremia and its association with disease severity^12^. A study that evaluated daily blood samples from 66 patients hospitalized with dengue fever until discharge found that daily viremia was higher in primary dengue fever compared to secondary dengue fever. The median viremia on the third day of illness was lower in secondary dengue fever^12^. A case report of vaccine-induced dengue fever, which occurred six days after the first dose of the TAK-003 vaccine, described a patient who had classic manifestations of dengue fever and elevated of inflammatory markers. Genome sequencing confirmed that the strain was vaccine-induced^13^.

Dengue outbreaks in Brazil were occurring across different regions at the time of this case, with serotypes DENV-1, DENV-2, and DENV-3 reported in all states. Epidemiological data indicated that severe dengue was predominantly associated with DENV-2^14^. The inability to sequence the virus was a limitation of our study, as it prevented us from defining the viral source. If this was a primary DENV-2 infection, our patient did not show any clinical or laboratory signs of disease severity, which was possibly due to the protection effect of immunization.

The use of live attenuated vaccines during large outbreaks poses challenges for clinical diagnostics and genomic surveillance, particularly in differentiating vaccine strains from wild-type strains. Notably, genomic sequencing has revealed high similarity between infecting strains and vaccine-derived strains, highlighting the diagnostic challenges in distinguishing between vaccine responses and wild-type infections during vaccination campaigns^15^. Although vaccination is crucial for protecting against severe forms of the disease, thorough evaluation of all cases remains necessary for prompt diagnosis and timely treatment.

## CONCLUSION

In conclusion, it is important to remember that atypical cases, particularly those without fever, require thorough investigation in regions where the vaccine is being introduced, so dengue can be excluded. Patients who have already had dengue would also benefit from the vaccine, as it offers protection against all four dengue virus serotypes and prior immunity to one serotype does not protect against the others. This reduces the risk of a second infection with a different serotype, which can be more severe, and offers more complete protection against the disease.

## Data Availability

The complete anonymized dataset supporting the findings of this study is included within the article itself.
